# Biochemical analyses demonstrate that Bt maize has no adverse effects on *Eisenia fetida*

**DOI:** 10.1371/journal.pone.0269303

**Published:** 2022-06-02

**Authors:** Fengci Wu, Zhilei Jiang, Baifeng Wang, Junqi Yin, Daming Wang, Xinyuan Song

**Affiliations:** Jilin Provincial Key Laboratory of Agricultural Biotechnology, Agro-Biotechnology Research Institute, Jilin Academy of Agricultural Sciences, Changchun, China; Universidade de Coimbra, PORTUGAL

## Abstract

The potential effects of Bt (*Bacillus thuringiensis*) maize on non-target organisms should be evaluated before such maize is commercially planted. Earthworms play an indispensable role in the soil ecosystem; act as important bio-indicators of soil quality and environmental pollution. Therefore, earthworms are often used as the object to evaluate the non-target effect of Bt maize. To accelerate the commercialization of transgenic maize in China, a 90-day *Eisenia fetida* feeding experiment was conducted to evaluate the potential effects of Bt maize line, BT799—which was developed by China Agricultural University and contains the *Cry1Ac* gene—and its non-Bt conventional isoline—Zheng 58—on *E*. *fetida*. Our results showed that the Bt maize line had no significant effects on the growth, reproduction, or enzymatic activities of these earthworms. In summary, Bt maize had no toxic effects on *E*. *fetida*.

## Introduction

China is a major producer of genetically modified crops, with Bt cotton being its main commercially produced transgenic crop. Bt maize is expected to become the second most popularised transgenic crop after Bt cotton, due to its important potential commercial application value. However, Bt maize releases Cry proteins into the soil ecosystem through pollen dispersion, stubble decomposition, root exudates [1] and straw return into the soil [2], possibly causing environmental risks. Hence, the potential impact of Bt maize on animals in the soil has been the focus of much scientific attention.

In recent years, laboratory study has become an important means to assess the effects of Bt plants on soil animal communities [3], and bio-indicators are key to the success of this method. Earthworms may reside at the bottom of the food chain, account for up to 90% of invertebrate biomass present in soil [4]. They promote the decomposition of organic matter by scavenging for decaying plant material in soil, increase nutrient cycling, and improve the physical and chemical properties of soil, thereby playing an essential role in the soil ecosystem [5–10]. Moreover, earthworms are directly exposed and display sensitivity to soil pollutants, making them useful markers to indicate and detect soil contamination, as well as to be applied in the ecological risk assessment of soil [5, 11, 12]. *E*. *fetida (Annelida*, *Lumbricidae)* is the most commonly used earthworm in soil ecotoxicological testing [13], and the OECD has established standardised methods for toxicity determination by this species [14, 15].

The Bt maize line, BT799, was developed by China Agricultural University and contains the *Cry1Ac* gene. Although several environmental risk studies have already been conducted on Cry proteins, including evaluation of the effects of transgenic *Cry1Ac* cotton on the earthworm [13], it is necessary to re-examine this protein in relation to BT799, to accelerate the commercialisation of this transgenic maize in China.

In this study, we used a laboratory method involving a 90-day feeding test on *E*. *fetida*, to evaluate the potential effects of the Bt maize line, BT799 and its non-Bt conventional isoline, Zheng 58. We not only evaluated the growth, development and reproduction of *E*. *fetida*, but also analysed the effects of Bt maize on the activities of catalase, peroxidase, superoxide dismutase and acetyl cholinesterase, in *E*. *fetida*.

## Materials and methods

### Plant materials

The transgenic Bt maize line, BT799 that contains *Cry1Ac* gene, and its corresponding non-transgenic isoline, Zheng 58, were provided for purposes of this study by China Agricultural University. Maturity stage plants (root, stem, leaf) from both lines were collected and dried at 60°C for 12 hours. Plant materials from the two lines were separately crushed and sifted through a 1.0 mm sieve before each batch was stored in a freezer at -20°C.

### Bio-indicators

The *E*. *fetida* test population is consistently reared in our laboratory. For the purposes of this study, 200 hermaphroditic earthworms were randomly selected and divided into two main treatment groups, which were subdivided into culture boxes.

### Experimental soil

The soil harbouring our *E*. *fetida* was collected from Gongzhuling City, Jilin Province, China. A soil layer of 5–25 cm in depth was collected from a source in which no transgenic plants had been planted. The soil was air-dried under natural conditions; thereafter plant residues, larger soil clumps and sundry particles were removed by passing the soil through a 15.0 mm sieve, followed by sifting through a 2.0 mm sieve. Finally, the soil was mixed well and set aside for further use.

### Feeding experiment

Previous research on returning straw to the field determined that the content of corn straw returned to the soil should not exceed 3.9% [16]; accordingly, we mixed the prepared plant material with the soil to match this guideline. After completely mixing each of the two plant material samples with their respective soil samples to compile two feeding treatments, 200 g of each mixture were placed in a square plastic container, with internal dimensions of 8.0 cm side length and 9.5 cm height, and an air hole in the cover. Ten *E*. *fetida* individuals—300–400 mg in weight and with reproductive rings—were selected, washed with distilled water, dried and weighed, and added to the plastic container. This process was repeated five times with the BT799-soil mixture, and five times with the Zheng 58-soil mixture. Each feeding treatment set thus consisted of five replicates, with ten earthworms being fed per container. The containers were placed in a climate chamber at 24 ± 1°C, at 65% relative humidity.

### Effect of Bt maize on growth and reproduction of *E*. *fetida*

Each maize-soil sample was considered as one feeding treatment. Each treatment set consisted of five replicates, and each replicate contained ten *E*. *Fetida* individuals that were fed in one plastic container. Observations were made on day 15, 30, 45, 60, 75, and 90 of the experiment. At each observation, the number of adult worms, young worms and cocoons in each culture box was counted and recorded. All living adult worms were washed with distilled water and weighed together, each time. Adult worms and cocoons were placed back into their original container to continue with the experiment, while young worms were removed.

### Effect of Bt maize on enzyme activity of *E*. *fetida*

We further set up five more replicates of each treatment, each replicate containing ten *E*. *fetida* individuals being fed in one plastic container. On days 15, 30, 45, 60, 75, and 90, 1–2 adult worms were selected from each plastic container, and combined with physiological saline at a weight (g):volume (ml) ratio of 1:9. The mixture containing *E*. *fetida* was pulped and centrifuged at 4,000 rpm for 10 minutes. The supernatant was extracted and again combined with nine times its volume of physiological saline. This 1% tissue homogenate was stored in a freezer at -80°C and used for the detection of enzyme activity. Catalase (CAT), peroxidase (POD), superoxide dismutase (SOD), and acetyl cholinesterase (AChE) activities were measured with ELISA test kits (A007, A084, A001, A024, Nanjing Jiancheng Bioengineering Institute, Nanjing, Jiangsu, China), and following the manufacturer’s instructions.

### Stability of Cry proteins in maize diet

The concentration of Cry proteins in the each earthworm feed was analysed by Bt-*Cry1Ab/1Ac* ELISA Kit (PSP 06200/0480, Agdia, USA) on samples of 2–3 mg of feed, on days 0, 15, 30, 45, 60, 75, and 90 of the enzyme activity experiment.

### Data analysis

The original data is sorted out by Excel (S1 Table), and two-way ANOVA (maize line* time) was performed.

## Results

### Stability of Cry proteins in maize diet

According to the results of ELISA measurements, the concentrations of *Cry1Ac* in the original Bt maize dietary matter were 241.87 ± 2.80 ng/g. After feeding exposure, *Cry1Ac* contents in the residual dietary matter decreased over time. On days 15, 30, 45, 60, 75, and 90, the concentrations of *Cry1Ac* in the feed samples were 74.46 ± 1.94 ng/g, 42.72 ± 1.39 ng/g, 23.06 ± 1.49 ng/g, 14.21 ± 0.47 ng/g, 10.98 ± 0.43 ng/g and 8.28 ± 0.24 ng/g, respectively. No Cry protein was detected in the feed prepared from non-Bt maize plants.

### Effect of Bt maize on the survival rate of *E*. *fetida*

**Fig 1:**
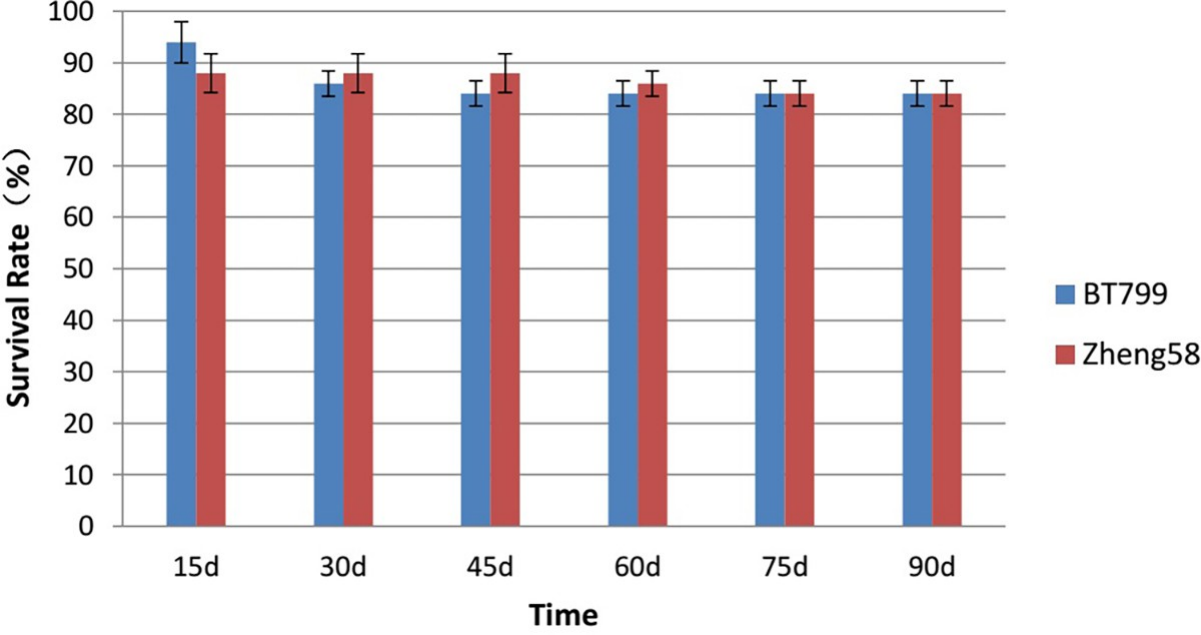
Effects of Bt maize on the survival rate of *E*. *fetida*, from six investigations. Values are means ± SE, n = 5.

Based on the outcome of ANOVA conducted on days 15, 30, 45, 60, 75, and 90 during the growth and reproduction experiment, it was revealed that the survival rates of *E*. *fetida* showed no significant difference between Bt and non-Bt feeding treatment groups (P = 0.847), and there were no significant differences between the two treatment groups at each time point (P>0.05) (Fig 1).

### Effect of Bt maize on the weight of *E*. *fetida*

**Fig 2:**
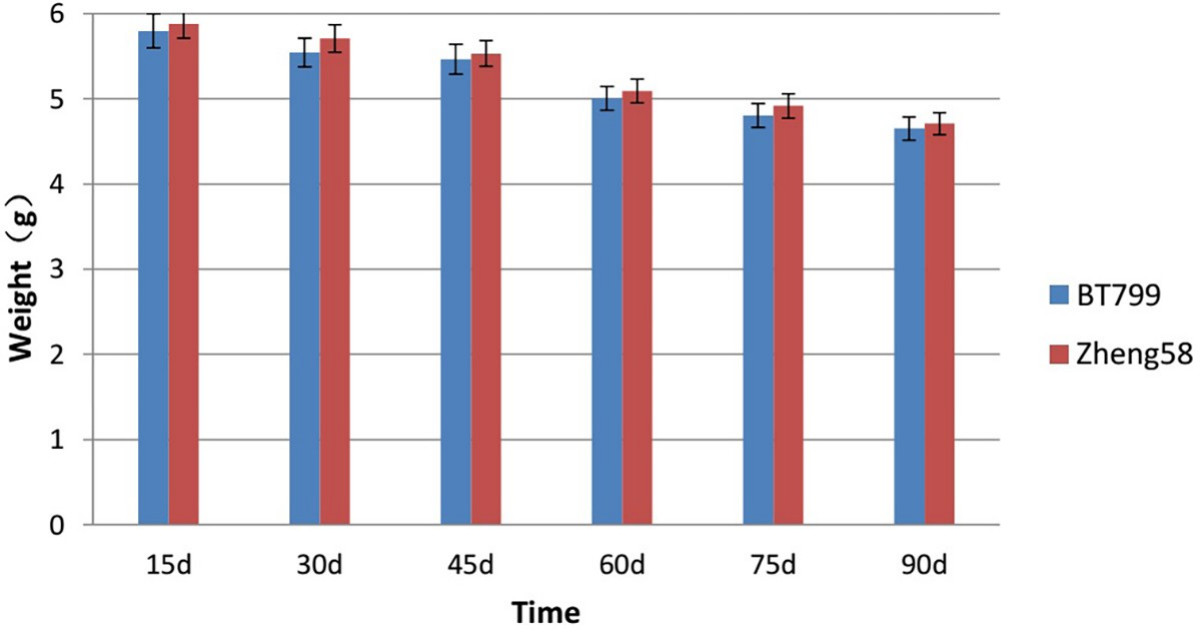
Effects of Bt maize on the weight of *E*. *fetida*, from six investigations. Values are means ± SE, n = 5.

Based on the outcome of ANOVA conducted on days 15, 30, 45, 60, 75, and 90 during the growth and reproduction experiment, no significant differences were perceived in the weight of *E*. *fetida* either between Bt and non-Bt feeding treatment groups (P = 0.295), or between the two treatment groups at each time point (P>0.05) (Fig 2).

### Effect of Bt maize on the reproduction of *E*. *fetida*

**Fig 3:**
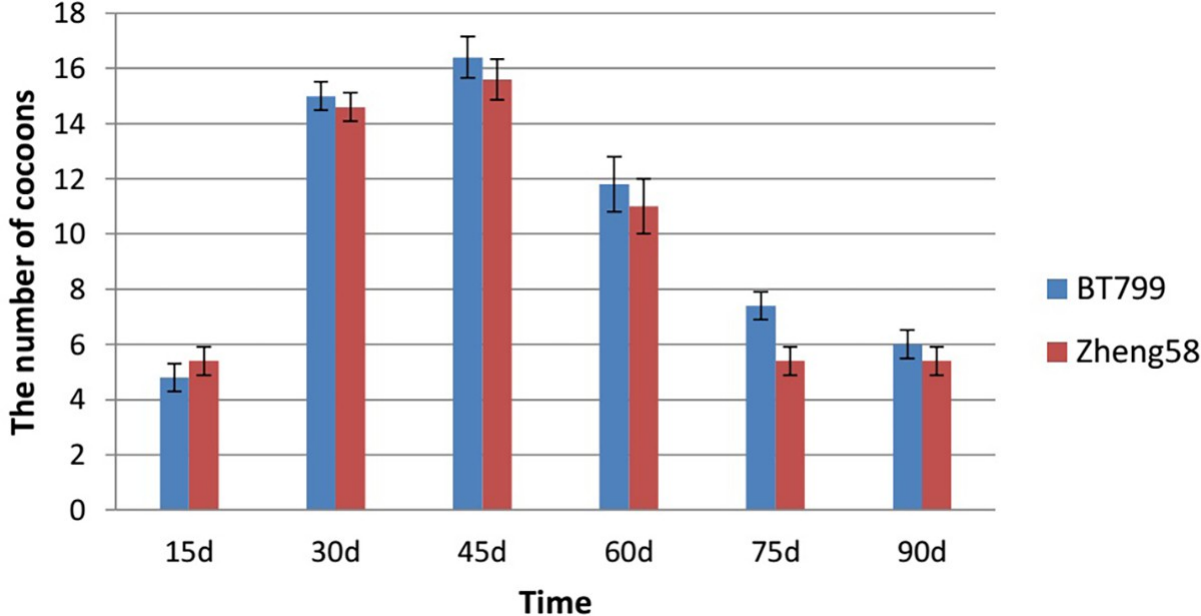
Effects of Bt maize on the number of cocoons produced by *E*. *fetida*. Values are means ± SE, n = 5.

Based on the outcome of ANOVA conducted on days 15, 30, 45, 60, 75, and 90 during the growth and reproduction experiment, there were no significant differences in the number of produced *E*. *fetida* cocoons between Bt and non-Bt feeding treatment groups (P = 0.080), and between the two treatment groups at each time point (P>0.05) (Fig 3).

**Fig 4:**
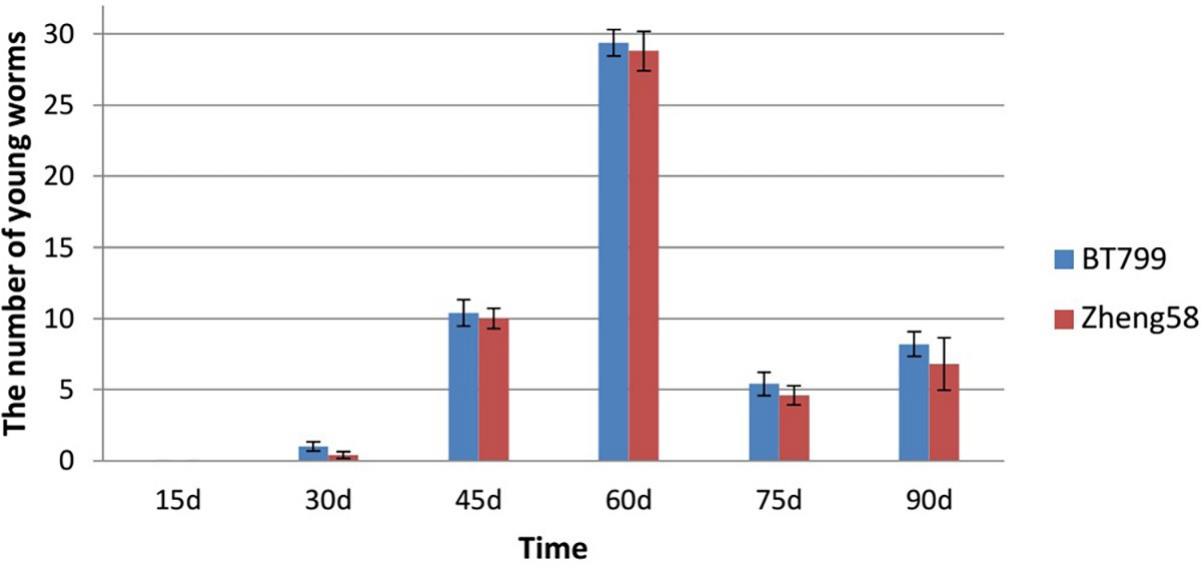
Effects of Bt maize on the number of young worms produced by *E*. *fetida*. Values are means ± SE, n = 5.

The same analysis method revealed no significant difference in the number of young *E*. *fetida* worms between the BT799 and Zheng 58 feeding treatment groups (P = 0.224); furthermore, there were no significant differences between the two treatment groups at each time point (P>0.05) (Fig 4).

### Effect of Bt maize on enzyme activity of *E*. *fetida*

Based on the outcome of ELISA conducted on days 15, 30, 45, 60, 75, and 90 during the Enzyme Activity experiment, no significant differences were observed in the activity levels of the four enzymes in *E*. *fetida*, that were fed with the two different diets (P>0.05). Furthermore, no significant differences in enzyme activity were observed between the two treatment groups at each time point (P>0.05) (Table 1).

**Table 1:** Enzyme activity of *E*. *fetida* fed with two different maize diets.

Enzyme	Day 15		Day 30		Day 45		Day 60		Day 75		Day 90		Total	
	BT799	Zheng 58	BT799	Zheng 58	BT799	Zheng 58	BT799	Zheng 58	BT799	Zheng 58	BT799	Zheng 58	BT799	Zheng 58
CAT U/mgprot	17.14±0.17a	17.25±0.13a	17.03±0.18a	17.01±0.11a	17.53±0.22a	17.36±0.12a	16.35±0.18a	16.61±0.07a	15.08±0.29a	15.83±0.32a	16.78±0.22a	16.20±0.29a	16.65±0.17a	16.71±0.13a
POD U/mgprot	21.58±0.22a	21.92±0.09a	17.32±0.24a	17.00±0.24a	27.70±0.42a	28.29±0.24a	28.31±0.26a	28.81±0.12a	27.62±0.19a	27.03±0.51a	29.37±0.22a	28.39±0.53a	25.32±0.82a	25.24±0.82a
SOD U/mgprot	61.82±0.37a	61.00±0.26a	50.89±0.39a	48.72±0.88a	41.36±0.37a	42.57±0.78a	28.75±0.65a	26.91±0.60a	22.71±0.60a	24.40±0.45a	24.21±0.32a	22.94±0.69a	38.29±2.69a	37.76±2.64a
AChE U/mgprot	4.48±0.02a	4.59±0.08a	4.34±0.04a	4.29±0.04a	4.19±0.05a	4.08±0.06a	4.11±0.02a	4.14±0.03a	4.44±0.07a	4.23±0.08a	4.35±0.06a	4.28±0.03a	4.32±0.03a	4.27±0.04a

CAT, catalase; POD, peroxidase; SOD, superoxide dismutase; AChE, acetyl cholinesterase

## Discussion

The binding of Cry proteins to specific receptors in target insects is an essential step in the intoxication process [17–19], which is key to the pesticidal functionality of these genes. Non-target organisms that do not possess the appropriate receptors, are therefore not sensitive to the specific toxins [20, 21]. Therefore, Bt plants generally do not have significant adverse effects on the growth and reproduction of non-target organisms. However, in order to ensure environmental safety, the evaluation of representative non target organisms is necessary. Verceri et al. [22] found that *Cry1Ab* protein from Bt corn leaves and root exudates had no effect on *Aporrectodea caliginosa* and Ahmad et al [23] demonstrated that Bt corn had no effect on the survival of *Lumbricus terrestris*. Moreover, many non-target animal species such as *Folsomia candida* [24–26], and *Micraspis discolor* [27], have been shown not to be affected by the Bt proteins expressed by transgenic Bt plants.

Earthworm is also often used as a species for evaluating the impact of Bt plants on non-target organisms. For example, Clark et al. [25] added Bt corn leaf powder to a mixture of horse manure and soil to feed *E*. *fetida*, and results showed that Bt corn had no harmful effects on the survival and reproduction of the earthworm; Liu et al. [13, 28] added crushed Bt cotton leaves to the soil used to feed *E*. *fetida*, with results showing no significant difference in the numbers of cocoons and young worms between the *E*. *fetida* group fed with Bt cotton leaves and the control; Shu et al. [29] simulated straw return under laboratory conditions, by feeding *E*. *fetida* with two types of Bt corn straw; The results showed that Bt corn straw had no significant impact on the survival of *E*. *fetida*. Kamota et al. [30] research shows that growing Bt maize does not have negative effects on the numbers of the earthworms in the Central Eastern Cape, South Africa. Shahid et al. [31] research shows that no lethal effects of transgenic Bt protein on the survival of earthworm.

In addition to growth and development indicators, the activities of enzymes in organisms are also used to evaluate the impact of Bt plants on non-target organisms. Several enzymes such as CAT, POD, SOD, and AChE, are important in insects response to pesticides and herbicides used in agriculture [32] and hence our study was partly aimed at determining the effect, if any, of Bt proteins on certain earthworm enzymes. Numerous similar studies have been previously conducted; Shu et al. [8] fed *E*. *fetida* with two types of transgenic corn, and the results showed that Bt corn treatment had no significant effect on the CAT activity of the earthworm. Cui [33] et al. conducted similar experiments and found no significant effects on AChE and CAT activity in earthworms. When Liu et al. [13] added crushed Bt cotton leaves to the soil used to feed *E*. *fetida*, results showed no significant differences in SOD activity in *E*. *fetida* fed with the Bt cotton line compared to those fed on a non-Bt cotton diet. In the study of Yuan et al. [34], both SOD and POD activity in *Folsomia candida* were not affected by *Cry1Ab* and *Cry1Ac* proteins. Bai et al. [26] also found no significant difference in SOD activity between the *Folsomia candida* that was fed with the leaves of Bt transgenic rice, as opposed to those fed with non-Bt rice leaves. Similarly, in this study we found no significant difference in the activity of CAT, POD, SOD, and AChE between the *E*. *fetida* fed on the leaves of Bt maize and those fed on non-Bt maize lines.

Bt protein can exist in soil for a long time and has activity. Research by Zwahlen et al. shows that the *Cry1Ab* protein can be retained in the field for at least 240 days [35]. Our research has been carried out a 90-day *E*. *fetida* feeding experiment, to quantify the exposure of earthworms to Cry protein during the feeding experiments, we used ELISA to determine the stability of *Cry1Ac* protein in the BT799 diet. Although the *Cry1Ac* concentrations in the dietary matter residues decreased over time, a certain level of *Cry1Ac* content remained detectable up to the end of the experiment, i.e., day 90. This indicates that *Eisenia foetida* has been exposed to Bt protein.

Our results showed that the Bt maize line had no significant effects on the growth, reproduction, or enzymatic activities of these earthworms. In summary, the planting of the Bt maize lines will pose a negligible risk to *E*. *fetida*.

## Supporting information

S1 Table Raw data from this study. (XLSX)
